# Metallic Nickel Nanoparticles May Exhibit Higher Carcinogenic Potential than Fine Particles in JB6 Cells

**DOI:** 10.1371/journal.pone.0092418

**Published:** 2014-04-01

**Authors:** Ruth Magaye, Qi Zhou, Linda Bowman, Baobo Zou, Guochuan Mao, Jin Xu, Vincent Castranova, Jinshun Zhao, Min Ding

**Affiliations:** 1 Public Health Department of Medical School, Zhejiang Provincial Key Laboratory of Pathological and Physiological Technology, Ningbo University, Ningbo, Zhejiang Province, People's Republic of China; 2 Pathology and Physiology Research Branch, Health Effects Laboratory Division, National Institute for Occupational Safety and Health, Morgantown, West Virginia, United States of America; Harvard Medical School, United States of America

## Abstract

While numerous studies have described the pathogenic and carcinogenic effects of nickel compounds, little has been done on the biological effects of metallic nickel. Moreover, the carcinogenetic potential of metallic nickel nanoparticles is unknown. Activator protein-1 (AP-1) and nuclear factor-κB (NF-κB) have been shown to play pivotal roles in tumor initiation, promotion, and progression. Mutation of the p53 tumor suppressor gene is considered to be one of the steps leading to the neoplastic state. The present study examines effects of metallic nickel fine and nanoparticles on tumor promoter or suppressor gene expressions as well as on cell transformation in JB6 cells. Our results demonstrate that metallic nickel nanoparticles caused higher activation of AP-1 and NF-κB, and a greater decrease of p53 transcription activity than fine particles. Western blot indicates that metallic nickel nanoparticles induced a higher level of protein expressions for R-Ras, c-myc, C-Jun, p65, and p50 in a time-dependent manner. In addition, both metallic nickel nano- and fine particles increased anchorage-independent colony formation in JB6 P^+^ cells in the soft agar assay. These results imply that metallic nickel fine and nanoparticles are both carcinogenetic *in vitro* in JB6 cells. Moreover, metallic nickel nanoparticles may exhibit higher carcinogenic potential, which suggests that precautionary measures should be taken in the use of nickel nanoparticles or its compounds in nanomedicine.

## Introduction

In recent years, nanotechnology has brought a lot of improvements and advances in the treatment and diagnosis of diseases due to the development of improved contrast agents [1], [2], and drug delivery vehicles [3], [4], using nanomaterials. The use of metallic nanoparticles and their compounds have been paramount in such advances. Nickel (Ni) and Ni compound nanoparticles are also among those being investigated, for example nickel zinc (NiZn) ferrite [6]. While many studies have been published on the carcinogenicity of nickel compounds [7], [8], [9], the carcinogenicity of metallic nickel is still uncertain [10], [11], [12]. In vivo experiments and epidemiology studies show that nickel compounds are carcinogenic [13]. Therefore, nickel compounds are classified by the International Agency for Research on Cancer (IARC) as carcinogens to human being [14]. Though the epidemiological studies on the carcinogenicity metallic nickel are not conclusive [15], experimental animal studies suggest that metallic nickel nanoparticles may be carcinogenic [10]. Nickel nanoparticles are widely used in industry due to its unique characteristics with production estimated to be around 20 tons per year in the United States [16]. However, concern has been expressed that these same properties of nickel nanoparticles may also present unique bioactivity and challenges to human health [17].

We have previously shown that metallic nickel nanoparticles induced higher up-regulation of phospho-Akt (protein kinase B) and Bcl-2 than fine particles [18]. Zhang *et al* found that metallic nickel nanoparticles had a much more toxic effect on the lung than metallic nickel fine particles, but the mechanism remains to be elucidated [19]. Roy *et al* reported that Akt proto-oncogene overexpression is an early event during sporadic colon carcinogenesis [20]. Therefore, up-regulation of proto-oncogenes may be important in the carcinogenicity of metallic nickel particles. Based on the fact that nanoparticles of metallic nickel and TiO_2_ cause more pronounced toxicity compared with their fine particles [18], [21], [22], [23], [24], particle size may be also an important factor for the carcinogenicity of metallic nickel particles [25]. Accordingly, an *in vitro* comparative study was performed on the carcinogenicity of metallic nickel nano- and fine particles.

## Materials and Methods

### Materials

Metallic nickel nanoparticles (average size 80 nm), fine particles (average size of 3 µm, Eagle's minimal essential medium (EMEM), Opti-MEM I medium, fetal bovine serum (FBS), trypsin, penicillin/streptomycin and L-glutamine, Alexa Fluor 488 donkey anti-goat antibody, antibodies, and luciferase assay substrate were purchased from the same companies as used in our previous study [18]. Protease inhibitor cocktail and 12-O-tetradecanoylphorbol-13-acetate (TPA) was purchased from Sigma Chemical Co. (St. Louis, MO).

### Methods

#### 1. Preparation of metallic nickel nano- and fine particles

Metallic nickel nano- and fine particles were prepared as in our previous study [18]. Briefly, under sterile conditions, stock solutions of metallic nickel nano- or fine particles were prepared by sonification on ice (Branson Ultrasonics Corp., Danbury, CT) in sterile PBS (10 mg/ml) for a total of 3 min at a power of 400 W with interval changes of 30 sec sonication and 15 sec on ice. Before use, these particles were diluted to a designed concentration in fresh culture medium.

#### 2. Size distribution and surface area measurements

The size distribution and surface area of metallic nickel particles were detected following the procedures used in our previous study [18]. Briefly, Surface area of metallic nickel particles was measured using the Gemini 2360 Surface Area Analyzer (Mircomeritics; Norcross, GA) with a flowing gas technique according to the manufacturer's instructions. The size distribution of metallic nickel particles was detected using scanning electron microscopy (SEM; Hitachi S-4800; Japan) according to the manufacturer's instructions. Optimas 6.5 image analysis software (Media Cybernetics; Bethesda, MD) was used to measure the diameter of metallic nickel particles.

#### 3. Cell culture

Mouse epidermal JB6 cells were obtained from Dr. Colburn' Lab (see reference No. 35) and cultured in 5% FBS EMEM supplemented with 2 mM L-glutamine and penicillin-streptomycin (10,000 U/ml penicillin and 10 mg/ml streptomycin) at 37°C (80% humidified air, and 5% CO_2_).

#### 4. Immunocytochemistry staining

JB6 cells were cultured in a 24-well plate until 60 to 80% confluent. Then, the cells were treated with metallic nickel fine or nanoparticles in 5% FBS EMEM for 24 h. Cells were fixed with 4% para-formaldehyde in PBS, permeabilized with 0.5% Triton X-100/PBS for 30 min, and blocked with 3% bovine serum albumin in PBS for 1 h at 37°C. The cells were then incubated with C-Jun or p65 antibody at 4°C overnight. After washing with PBS, Alexa Fluor 488 secondary antibody was added. Cells were incubated at room temperature for 2 h. The cells were then washed twice with PBS, and resuspended in fresh PBS (0.2 ml/well). Fluorescence images were acquired with an immunofluorescence microscope.

#### 5. Western blot analysis

Briefly, cells were plated onto a 6-well plate. The cultures were grown 24 h and then starved in 0.1% FBS EMEM overnight. Cells were treated with metallic nickel nano- or fine particles for 1, 3, 6 or 8 h, respectively. After treatment, western blot was conducted as stated in our previous study [18]. Immunoblots for expressions of R-Ras, c-myc, C-Jun, p65, p50, p53, HIF-1alpha and beta tubulin were detected. Experiments were performed three or more times. Equal loading of protein was conducted by measuring beta tubulin expression.

#### 6. Detection of AP-1, NF-κB or p53 transcription activity

JB6 cells, stably transfected with the AP-1, NF-κB or p53-luciferase plasmid as described previously [26] were seeded onto a 24-well plate. Cells (5×10^4^ in 1 ml of culture medium) were grown 12 h and then starved in 0.1% FBS EMEM overnight. Then, cells were exposed to different concentrations of metallic nickel fine or nanoparticles (0–20 µg/cm^2^) for 24 h. Thereafter, cells were lysed with 100 µl of luciferase lysis buffer. The luciferase activity of AP-1, NF-κB or p53 was measured by addition of luciferase assay substrate using a luminometer (Monolight 3010, Analytical Luminescence Laboratory, San Diego, CA). The results were expressed as relative AP-1, NF-κB or p53 luciferase activity compared with untreated controls.

#### 7. Anchorage-independent transformation assay

The effect of metallic nickel fine or nanoparticles on cell anchorage-independent transformation was evaluated in a JB6 P^+^ cell line using the soft agar assay as described previously [27]. JB6 P^+^ cells were treated with/without metallic nickel nano- or fine particles (1 µg/cm^2^) until only a few live cells remained (approximately three weeks). Live cells were then allowed to grow until confluent in a 6-well plate, then the treatment, as stated above, was repeated for a total of 3 times. Cells were then collected and stored in liquid nitrogen until ready to use. Metallic nickel nano- or fine particle-treated or non-treated JB6 P^+^ cells (1×10^4^) were incubated on soft agar medium. The cultures were maintained in a humidified, 37°C, 5% CO_2_ incubator for 21 days. The anchorage-independent colonies were counted as described previously [28], [29]. 12-O-tetradecanoylphorbol-13-acetate (TPA, 20 ng/ml) was set as a positive control.

#### 8. Statistical analysis

Significant differences were determined using R software or the Student's *t*-test.

## Results

### 1. Size distribution and surface area of metallic nickel particles

To measure the size distribution and surface area of nickel particles, scanning electron microscopy and Gemini 2360 Surface Area Analyzer were used, respectively. The size distribution of metallic nickel fine and nanoparticles is 3.3 µm and 92.3 nm, respectively. The average surface area of metallic nickel fine and nanoparticles are 0.4 m^2^/g and 4.0 m^2^/g, respectively.

### 2. Nuclear translocation of c-Jun and alteration of AP-1 activity after metallic nickel particle exposure

**Figure 1:**
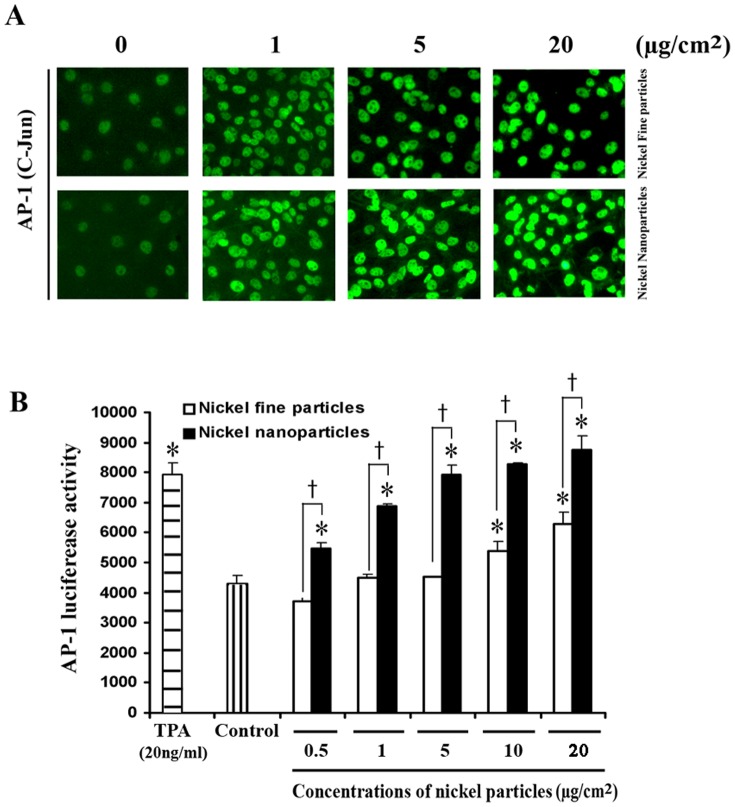
Induction of c-Jun nuclear translocation and AP-1 activation by metallic nickel particles. JB6 cells, stably transfected with AP-1 luciferase reporter plasmid, were seeded onto 24-well plate and incubated overnight. Cells were treated with/without metallic nickel nano- or fine particles for 24 h. (A) Effect of metallic nickel particles on nuclear translocation of the c-Jun subunit of AP-1 as measured by indirect immunofluorescence analysis using an anti-c-Jun primary antibody. The bright greenish areas represent nuclei that stained with anti-c-Jun antibody. After treatments, cells were fixed, permeabilized, blocked, and stained with monoclonal c-Jun antibody for 3 h. Alexa Fluor 488 goat anti-rabbit secondary antibody was added for 1 h. AP-1 is 100% expression in all cell nuclei, metallic nickel nano- or fine particles may increase the expression (staining density) in the cell nucleus. (B) The AP-1 activity was measured by the luciferase activity assay. Results are means and standard errors of four assay wells. The experiment was repeated two times. * *P*<0.05 versus control; † *P*<0.05 versus fine particles. TPA (20 ng/ml) was set as a positive control.

To determine the change of nuclear translocation of c-Jun and the alteration of transcriptional activity of AP-1 following exposure of metallic nickel particles, immunocytochemistry staining and luciferase activity assay were performed. Immunocytochemistry staining (Figure 1A) shows that c-Jun was significantly translocated into the nuclei in cells after 24 h treatment with metallic nickel nano- or fine particles compared to control. Luciferase activity assay shows that both metallic nickel fine and nanoparticles induced AP-1 transactivation with a dose-dependent manner. The nanoparticles showed a significant higher AP-1 activation than fine particles (Figure 1B).

### 3. Nuclear translocation of p65 and alteration of NF-κB activity after metallic nickel particle exposure

**Figure 2:**
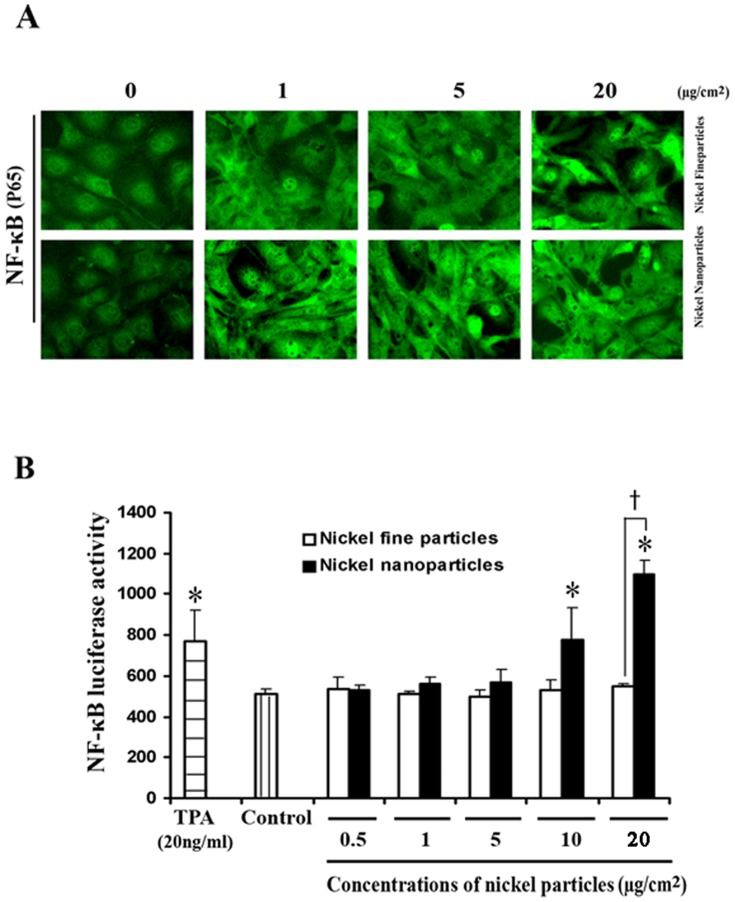
Induction of p65 nuclear translocation and NF-κB activation by metallic nickel particles. JB6 cells, stably transfected with NF-κB luciferase reporter plasmid, were seeded onto 24-well plate and incubated overnight. Cells were treated with/without metallic nickel nano- or fine particles for 24 h. (A) Effect of nickel particles on nuclear translocation of the p65 subunit of NF-κB as measured by indirect immunofluorescence analysis using an anti-p65 primary antibody. The bright greenish areas represent nuclei that stained with anti-p65 antibody. After treatments, cells were fixed, permeabilized, blocked, and stained with NF-κB monoclonal p65 antibody for 3 h. Alexa Fluor 488 goat anti-rabbit secondary antibody was added for 1 h. NF-κB is 100% expression in all cell cytoplasm, metallic nickel nano- or fine particles may induce NF-κB translocation from cytoplasm into the cell nucleus. (B) The NF-κB activity was measured by the luciferase activity assay. Results are mean and standard error of four assay wells. The experiment was repeated two times. * *P*<0.05 versus control; † *P*<0.05 versus fine particles. TPA (20 ng/ml) was set as a positive control.

To determine the changes in nuclear translocation of p65 and alteration of transcriptional activity of NF-κB following exposure of metallic nickel particles, immunochemistry staining and luciferase activity assay were performed. Immunocytochemistry staining (Figure 2A) shows that p65 was significantly transloctaed into the nuclei in cells after 24 h treatment with metallic nickel nano- or fine particles compared to control. Luciferase activity assay show that both metallic nickel fine and nanoparticles induced NF-κB transactivation with a dose-dependent manner. NF-κB transcription activity induced by nanoparticles was significantly higher than that induced by fine particles (Figure 2B).

### 4. Effects of metallic nickel particles on p53 activity and protein levels

**Figure 3:**
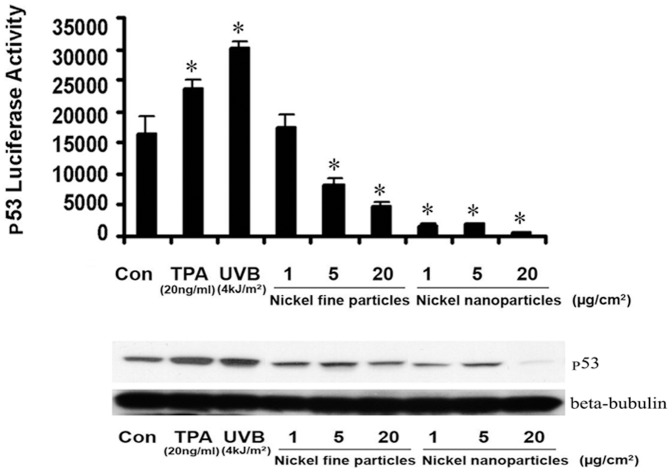
Effects of metallic nickel particles on p53 transcription activity and protein levels. JB6 cells, stably transfected with p53 luciferase reporter plasmid, were seeded onto a 24-well plate and incubated overnight. Cells were treated with/without metallic nickel nano- or fine particles for 24 h. The p53 transcription activity and protein levels were measured by the luciferase activity assay (A) and western-blot (B), respectively. Metallic nickel nanoparticles caused a greater decrease of p53 transcription activity and protein levels than fine particles. * *P*<0.05 *versus* control. TPA (20 ng/ml) or UVB (4 kJ/m^2^) were set as a positive control.

To study the effect of metallic nickel particles on p53 transcriptional activity, a JB6 cell line carrying a stably transfected p53-luciferase reporter plasmid was used. The cells were exposed to various doses of metallic nickel nano- or fine particles (1–20 µg/cm^2^) for 24 h, and then the luciferase activity was determined. The results show that both metallic nickel nano- and fine particles significantly decreased p53 transcription activity in a dose-dependent manner (Figure 3A). The maximum decrease of p53 transcription activity occurred at the concentration of metallic nickel particles between 5 and 20 µg/cm^2^. p53 protein levels were detected by western-blot (Figure 3B).

### 5. Effects of metallic nickel particles on R-Ras, c-myc, c-Jun, p65, p50, and HIF-1α expression

**Figure 4:**
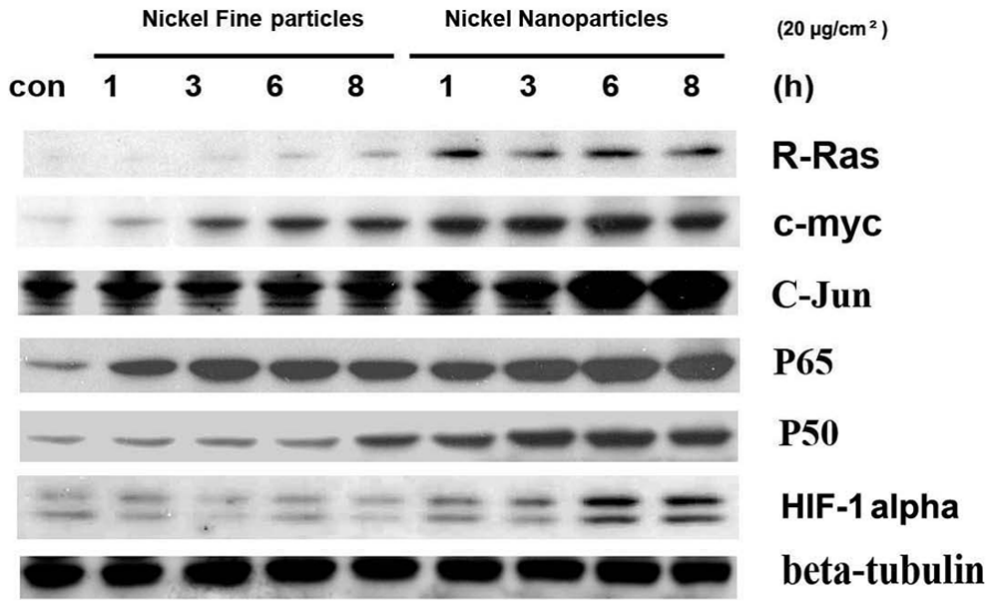
Effects of metallic nickel particles on protein expressions of R-Ras, c-myc, c-Jun, p65, p50 and HIF-1alpha. JB6 cells were treated with 20 µg/cm^2^ metallic nickel nano- or fine particles for 1, 3, 6 and 8 h. Western blot results show that both metallic nickel nano- and fine particles induced up-regulation of protein expressions of R-Ras, c-myc, c-Jun, p65, and p50 in a time-dependent manner. Metallic nickel nanoparticles elicited a higher stimulation on these protein expressions compared to fine particles. Nanoparticles induced a significant HIF-1alpha up-regulation after 6 and 8 h treatment.

To investigate the effects of metallic nickel particles on protein expressions of R-Ras, c-myc, c-Jun, p65, p50, and HIF-1α, western blot was performed. Metallic nickel fine or nanoparticles induced up-regulation of protein expressions of R-Ras, c-myc, c-Jun, p65, and p50 in a time-dependent manner. In addition, levels of these protein expressions induced by nanoparticles were significantly higher than that induced by fine particles. Nanoparticles induced a significant HIF-1α up-regulation after 6 and 8 h treatment (Figure 4).

### 6. Effects of metallic nickel particles on anchorage-independent colony formation

**Figure 5:**
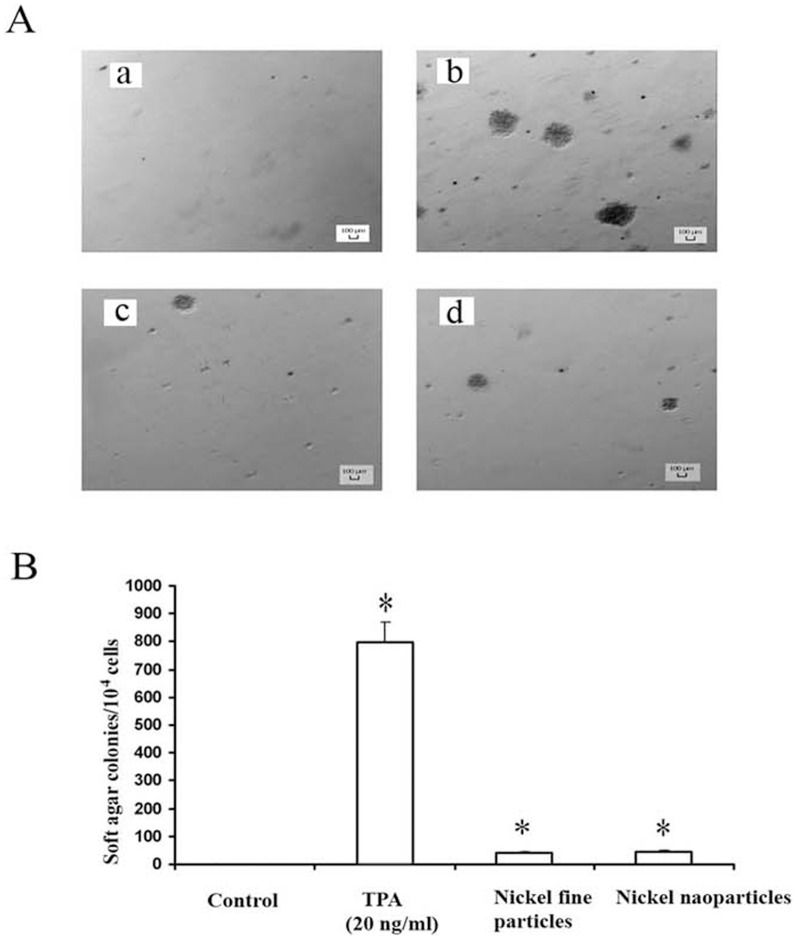
Effects of metallic nickel particles on colony formation of JB6 P^+^ cells in soft agar assay. Metallic nickel nano- or fine particle-treated or untreated JB6 P^+^ cells (1×10^4^) were incubated on soft agar medium for 21 days. TPA (20 ng/ml) was set as a positive control. (A) Images of colony formation of JB6 P^+^ cells in soft agar assay. (a) Control. (b) TPA (20 ng/ml). (c) Metallic nickel fine particles. (d) Metallic nickel nanoparticles. (B) Number of colonies/10^4^ cells in soft agar assay. The cell colonies were scored by a computerized image analyzer. * *P*<0.05 versus untreated control.

Based on activation of metallic nickel particles on tumor promoter AP-1 and NF-κB, our next aim was to determine whether metallic nickel particles induce JB6 P^+^ cell transformation. Cells (1×10^4^) pretreated with metallic nickel nano- or fine particles as stated in the materials and methods were evaluated in a soft agar assay for 21 days. The colonies formed in the soft agar were recorded. The results indicate that both metallic nickel nano- and fine particles induced JB6 P^+^ cell transformation. However, no significant difference in colony transformation rates was found between nano- and fine particle-treated cells (Figure 5).

## Discussion

Nickel is a widely distributed metal. The high consumption of various nickel products inevitably leads to occupational and environmental pollution [30]. In occupational settings, about 10% of all the primary nickel produced is used in metallic form [10]. Human exposure to nickel or its compounds has the potential to produce a variety of pathological effects. The major health effect of nickel exposure are skin allergies, lung fibrosis, and lung cancer [14]. Numerous studies have documented the carcinogenicity of nickel compounds [7], [8], [9], but the carcinogenic effect of metallic nickel remains to be clarified. This paper provides evidence that metallic nickel is carcinogenic with nanoparticles being more carcinogenic than fine particles.

Toxicity of particles is not only related to exposure intensity but also to the particle size [18]. Accordingly, more and more concern has been raised on the health effects of metallic nickel nanoparticles with their increase use in modern industries [31].

Our previous study shows that metallic nickel nanoparticles were more toxic than fine particles. Metallic nickel nanoparticles induced higher activation of phospho-Akt and Bcl-2 than fine particles [18]. The physicochemical properties of metallic nickel nanoparticles differ from their bulk material of the same composition. However, little is known about the underlying effects of metallic nickel nanoparticles in carcinogenesis. In the present study, the potential carcinogenesis of metallic nickel fine and nanoparticles was compared in a JB6 cell line *in vitro*. The JB6 cell line is derived from primary cultures of neonatal BALB/c epidermal cells and is suitable for studying differential responses to tumor promoters.

Several cellular signaling transcription factors including AP-1 and NF-κB have been shown to play pivotal roles in tumor initiation, promotion, and progression [32]. Activated AP-1 and NF-κB are found in many different cancer cells [33] and may result in the expression of target genes that are involved in neoplastic transformation and tumor progression [34]. On the contrary, inhibition of AP-1 and NF-κB activation has been shown to reduce neoplastic transformation [35]. Studies in transgenic mice indicate that AP-1 transactivation is required for tumor promotion [35]. In addition, the signal transduction pathways of AP-1 and/or NF-κB are known to be important molecular targets of chemo-preventive strategies [36]. Our present results show that both metallic nickel nano- and fine particles induced a dose-related increase of AP-1 and NF-κB transcription activity in JB6 cells after 24 h exposure. Furthermore, metallic nickel nanoparticles elicited stronger stimulation on transactivation of AP-1 or NF-κB than fine particles. Immunocytochemistry staining indicates that both c-Jun (subunit of AP-1) and p65 (subunit of NF-κB) were significantly translocated into the nuclei in cells after 24 h treatment with metallic nickel fine or nanoparticles. Luciferase activity assay shows that both metallic nickel nano- and fine particles significantly induced AP-1 and NF-κB activation with a dose-dependent manner. Moreover, AP-1 or NF-κB activity induced by nanoparticles was significantly higher than fine particles within a certain dose range (see Figure 1B and Figure 2B). This is in agreement with our previous study, which indicated that metallic nickel nanoparticles elicited higher activation of tumor promotion factors phospho-Akt and Bcl-2 than fine particles [18]. Based on these results, metallic nickel nanoparticles may be higher carcinogenic than fine particles. It should be noted that surface area of metallic nickel nanoparticles is 10 times greater than fine particles, but increase of AP-1 or NF-κB transcription activity induced by metallic nickel nanoparticles was somewhat less than 11-fold greater than that induced by fine particles. Therefore, bioactivity of metallic nickel particles may not be proportional to their surface area. The human tumor suppressor protein p53 is a key regulator of the response to cellular stresses such as DNA damage, nucleotide depletion, mitogenic oncogenes, and the tumor micro-environmental stress of hypoxia [37], [38]. The importance of p53 protein may be due to its effect on cell cycle progression, including cell proliferation and apoptosis [39], [40]. A study by Wong *et al* showed that Ni (II) is a strong activator of p53 protein in H460 human lung epithelial cells [41]. They also stated that Ni(II)- activated p53 triggers caspase 9/3-mediated apoptotic program and that it is an independent stress response to hypoxia- mimicking Ni (II). However our results show that p53 activation was decreased in JB6 cells treated with Ni nanoparticles. This shows that in JB6 cells treated with Ni nanoparticles, p53 activation maybe be suppressed due to increased growth factor binding and stress response genes such as HIF-1α. Cosse *et al* found that a decrease in p53 protein level and an increase in c-Jun DNA binding activity induced by hypoxia results in cancer cell resistance to anti-cancer drugs [42]. In addition, Vormer *et al* reported that partial ablation of the p53 pathway strongly stimulated anchorage-independent growth of mouse embryonic fibroblasts [43], while the mutation of the p53 tumor suppressor gene is considered to be one of the steps leading to the neoplastic state [40].

Ras and c-myc are tumor promoter genes. Ras gene products are involved in regulating cell growth and differentiation. Over expression and amplification of the Ras gene can lead to continuous cell proliferation, which is a major step in the development of cancer [44]. Dysregulated expression of c-myc oncogene is also associated with many malignancies [45]. Hypoxia can modulate the activity of many transcription factors, such as NF-κB, p53, c-myc, c-Jun, ETS-1, Egr-1, or SP-1 [46], [47]. In the present study, metallic nickel particles instigated a similar bioactivity as hypoxia in JB6 cells, which include a decrease of p53 and an increase of AP-1 and NF-κB trans- activation. Western blots show that both metallic nickel nano- and fine particles induced an increase of protein expressions of R-Ras, c-myc, c-Jun, p65, and p50 in a time-dependent manner. Nanoparticles induced a higher level of the expression of these proteins than fine particles.

HIF-1 is composed of HIF-1α and HIF-1β. The production of HIF-1α has been identified as a key element in allowing cells to adapt and survive in a hostile hypoxic environment via a variety of pathways [48]. In the present study, only nanoparticles but not fine particles induced significant HIF-1α up-regulation after 6 and 8 h treatment. These results suggest that further studies are needed for clarifying the roles of hypoxia-inducible signaling pathway in metallic nickel particle-induced carcinogenic process.

Anchorage independent growth is one of the hallmarks of transformation and is considered to be the most accurate and stringent *in vitro* assay for determining malignant transformation of cells [43]. Earlier studies have indicated that activation of AP-1 and/or NF-κB is essential for the neoplastic transformation of JB6 cells [34], [35], [49]. Inhibition of c-Jun or AP-1 represses transformation of JB6 cells [50]. Thus, AP-1 and NF-κB transcriptional activation instigated by metallic nickel particles may lead to cell proliferation and neoplastic transformation. To test this hypothesis, mouse epidermal JB6 P^+^ cells were used as an *in vitro* model of skin carcinogenesis. Our results demonstrate that both metallic nickel nano- and fine particles induced JB6 P^+^ cell transformation compared to control. Interestingly, no significant difference of colony transformation rate was found between metallic nickel nano- and fine particle-treated cells.

**Figure 6:**
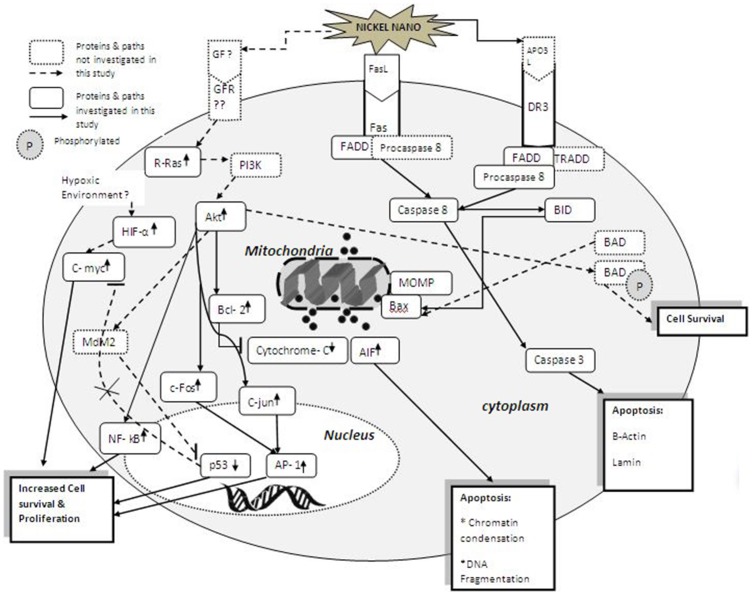
A summative diagram of the potential pathways involved in nickel nanoparticles induced apoptosis and carcinogenesis in JB6 cell line.

Taken together, the major findings of the present study include that metallic nickel nanoparticles induced higher activation of AP-1 and NF-κB than fine particles. We also identified that metallic nickel nanoparticles caused a greater decrease of p53 transcription activity and protein level than fine particles, implying that increased growth factor receptor binding or increased hypoxic environmental conditions may play a role in Ni nanoparticle suppression of p53. We provided evidence that metallic nickel nanoparticles induced a higher level of protein expressions for R-Ras, c-myc, C-Jun, p65, and p50 in a time-dependent manner. We demonstrated that both metallic nickel nano- and fine particles increased anchorage-independent colony formation in JB6 P^+^ cells in the soft agar assay. These results imply both metallic nickel fine and nanoparticles are carcinogenetic *in vitro*, however, metallic nickel nanoparticles may exhibit higher carcinogenic potential. Activation of AP-1 and NF-κB, up-regulation of R-Ras and c-myc protein expressions, as well as increase of p65 transcription activity may all contribute to the carcinogenicity of metallic nickel particles. Figure 6 shows a summative diagram of the possible pathways involved in nickel nanoparticles induced apoptosis and carcinogenesis from our previous and current study on the effects of nickel nanoparticles on JB6 cells. It shows that in JB6 cells nickel nanoparticles may activate the intrinsic pathway by stimulating the death receptor Fas (CD95/Apo1) and death receptor 3 (DR3). The Fas ligand (FasL), a homotrimeric protein acts as a ligand for Fas and causes oligomerization of its receptor on binding, leading to clustering of the death domains and binding of the cofactor Fas-associated via death domain (FADD). The FADD protein binds *via* its death-effector domain (DED) motif to a homologous motif in procaspase 8, forming the death inducing signaling complex (DISC). Upon recruitment by FADD, procaspase 8 oligomerization drives its activation through self-cleavage. Apo3 ligand (Apo3L) acts as a ligand for DR3 leading to binding of the cofactor TNFR1-associated death domain (TRADD) and activates procaspase 8. Activated Caspase 8 activates Caspase 3 through two pathways. In the first pathway Caspase 8 causes cleavage of procaspase 3 forming Caspase 3. Caspase 3 then activates β-actin and lamin leading to apoptosis. In the second pathway, Caspase 8 cleaves BID (Bcl-2 interacting protein) and its COOH-terminal part translocates to the mitochondria where it triggers the formation of mitochondrial outer membrane pores (MOMP) by activating Bax. The formation of MOMP releases the apoptotic factors cytochrome-c and apoptosis inducing factor (AIF). The release of AIF leads to chromatin condensation and DNA fragmentation. However in this study the release of cytochrome-c was inhibited by BCl-2. The inhibition of cytochrome-c release by Bcl-2 suggests that nickel nanoparticles caused apoptosis by Caspase 8/AIF cytochrome-c independent pathway. Nickel nanoparticles may also stimulate the binding of growth factors to the growth factor receptor leading to increased activation of Akt in a PI3K dependent manner through R-Ras [51]. Increased Akt activation leads to increased expression of Bcl-2 protein. Akt could also activate phosphorylation of the pro-apoptotic protein BAD, leading to cell survival, or inactivating it's prosphorylation leading to apoptosis. Akt also causes cell survival by increasing the activation of NF-kB and AP-1. The transcription factor AP-1 is activated via the JNK pathway and the subsequent activation of the c-Fos and c-Jun proteins which combine to form the AP-1 dimer. The decreased p53 expression may also be caused by the increased expression of Akt which binds and phosphorylates the MDM2 protein which ubiquitinates p53. In addition to receptor-mediated apoptosis, intrinsic apoptotic pathway can be activated by various forms of cellular stress such as hypoxia. Nickel nanoparticles may have caused hypoxic environment in the cell leading to increased expression of HIF-1α, causing increased activation of the c-myc protein [47]. C-myc is further increased in expression due to the lack of feedback inhibition by p53. Our study also shows a need for further investigation of the pathways where p53 is involved and the events associated in its decreased expression. A decreased apoptotic stimuli and increased stimulus for cell proliferation, could eventually lead to tumorigenic progression of JB6 cells treated with nickel nanoparticles. The results obtained from this study will also be of benefit for elucidating the pathogenic and carcinogenic potential of metallic nickel particles. In addition, the results may be useful as a reference when comparing the carcinogenic potential of different nickel compounds.
